# Enhanced Field Emission and Low-Pressure Hydrogen Sensing Properties from Al–N-Co-Doped ZnO Nanorods

**DOI:** 10.3390/nano14100863

**Published:** 2024-05-16

**Authors:** Youqing Tu, Weijin Qian, Mingliang Dong, Guitao Chen, Youlong Quan, Weijun Huang, Changkun Dong

**Affiliations:** Wenzhou Key Lab of Micro-Nano Optoelectronic Devices, Wenzhou University, Wenzhou 325035, China; yookingtu@163.com (Y.T.); dml13946319580@126.com (M.D.); 13124983550@163.com (G.C.); 17377260603@163.com (Y.Q.); 18857757816@163.com (W.H.)

**Keywords:** ZnO nanorods, Al–N-co-doped, hydrothermal method, field emission, low pressure, hydrogen sensing

## Abstract

ZnO nanostructures show great potential in hydrogen sensing at atmospheric conditions for good gas adsorption abilities. However, there is less research on low-pressure hydrogen sensing performance due to its low concentration and in-homogeneous distributions under low-pressure environments. Here, we report the low-pressure hydrogen sensing by the construction of Al–N-co-doped ZnO nanorods based on the adsorption-induced field emission enhancement effect in the pressure range of 10^−7^ to 10^−3^ Pa. The investigation indicates that the Al–N-co-doped ZnO sample is the most sensitive to low-pressure hydrogen sensing among all ZnO samples, with the highest sensing current increase of 140% for 5 min emission. In addition, the increased amplitude of sensing current for the Al–N-co-doped ZnO sample could reach 75% at the pressure 7 × 10^−3^ Pa for 1 min emission. This work not only expands the hydrogen sensing applications to the co-doped ZnO nanomaterials, but also provides a promising approach to develop field emission cathodes with strong low-pressure hydrogen sensing effect.

## 1. Introduction

Hydrogen is widely used in industrial production and energy fields, but the leakage or residue of hydrogen gas may bring safety hazards [1]. For example, the leakage of hydrogen could lead to an explosion with its concentration in a wide range of 4.0 to 75.6%. When hydrogen diffuses into the metal lattice, the strength of metal materials will be weakened. In addition, the residual hydrogen gas in vacuum electronic devices may affect the service life of the devices seriously. Therefore, the detection of hydrogen is very significant in many application fields [2]. At present, most research focuses on hydrogen detection at atmospheric pressure, and there are few researches on low-pressure hydrogen sensing [3,4,5,6,7,8]. Huang et al. reported a nanomechanical beam resonant hydrogen sensor operated in the pressure of 10^−5^−10^−4^ Torr [3]. Our groups developed a field emission hydrogen sensing technology based on carbon nanotubes, which extend the test pressure down to 10^−7^ to 10^−3^ Pa range [4].

Zinc oxide (ZnO), a typical n-type semiconductor material with a direct bandgap of 3.37 eV, has become an important field emission material due to its excellent thermal stability and oxidation resistance. Many methods have been used to synthesis ZnO nanorods, such as hydrothermal method [9], electrodeposition method [10] and chemical vapor deposition [11], etc. Among them, the low-temperature hydrothermal method has the advantages of low cost, simple operation, and large-scale growth, which has been widely used to prepare other metal oxides [12,13]. This material also presented excellent hydrogen gas sensing properties in atmospheric pressure environments [14]. Recently, doping or co-doping ZnO with various elements can effectively adjust the electronic structure, thereby enhancing the hydrogen sensing response [15,16,17,18,19]. Jaballah et al. reported that Al–Mg co-doped ZnO nanoparticles exhibited the best sensing performance, showing a fast and high response towards 2000 ppm hydrogen at 250 °C [18]. Al-Asedy et al. demonstrated that the gas sensing efficiency of the Al–Ga co-doped ZnO sample increased with the increase of the temperature and H_2_ concentration [19]. Despite these progresses, the low pressure hydrogen sensing performances for ZnO-based nanomaterials are still not investigated.

Herein, in this paper, Al–N-co-doped zinc oxide nanorods were successfully synthesized on the Si substrate for low-pressure hydrogen sensing. The results indicate that the Al–N-co-doped ZnO sample showed the best hydrogen sensing performance among all ZnO samples with the current increase of 140% for 5 min emission. In addition, the increase in the amplitude of the sensing current for the Al–N-co-doped ZnO sample could reach 75% at the pressure 7 × 10^−3^ Pa during a 1-min emission. The results indicate that the construction of Al–N-co-doped ZnO nanorods is able to improve effectively the hydrogen sensing performance of the ZnO nanorods.

## 2. Materials and Methods

**Figure 1:**
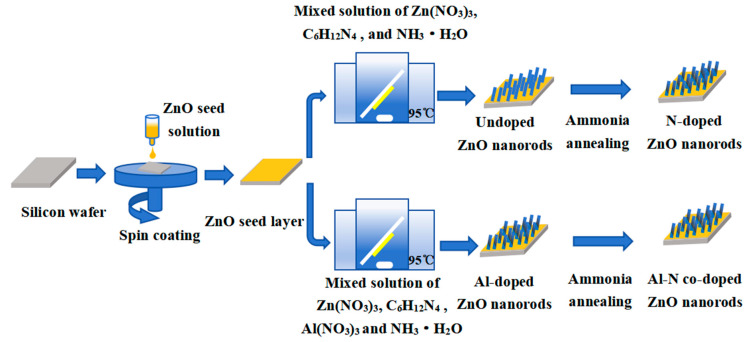
Preparation process diagram of the undoped, Al-doped, N-doped, and Al–N-co-doped ZnO nanorods.

The preparation process diagram of the undoped, Al-doped, N-doped, and Al–N-co-doped ZnO nanorods is shown in Figure 1.

### 2.1. Synthesis of ZnO Seed Films

ZnO nanorods were prepared on Si substrate using a low-temperature hydrothermal method [20]. The first step is to grow a layer of ZnO seed on the Si substrate. ZnO seed solution was obtained by mixing Zn(CH_3_COO)_2_ (0.02 M) methanol solution with NaOH methanol solution (0.03 M) at 60 °C for 2 h. Then ZnO seed solution was coated on the cleaned Si substrate after ultrasonic treatment. Finally, the Si substrate coated with ZnO seed was annealed at 350 °C for 30 min to enhance the adherence between the ZnO seed and the substrate.

### 2.2. Synthesis of ZnO Nanorods

Firstly, the aqueous solutions of Zn(NO_3_)_3_·6H_2_O (0.036 mol·L^−1^) and C_6_H_12_N_4_ (0.036 mol·L^−1^) were mixed together, then 7.5 mL of ammonia (25%) was added to the mixed solution. In the next step, the Si substrates coated with a thin ZnO seed layer were immersed in the mixed solution. Finally, undoped ZnO nanorods were grown successfully on the Si substrates with a reaction temperature of 95 °C for 6 h.

### 2.3. Synthesis of Al, N-Doped, and Al–N-Co-Doped ZnO Nanorods

Al-doped ZnO nanorods can be easily obtained by adding Al(NO_3_)_3_·9H_2_O (0.008 mol·L^−1^) into the mixed solution (Zn(NO_3_)_3_·6H_2_O and C_6_H_12_N_4_), while other reaction conditions remain unchanged. Then, the as-prepared samples were annealed at 300 °C for 60 min with the argon of 100 sccm. N-doped ZnO nanorods can be obtained through annealing with ammonia (15 sccm) with the Ar flow (140 sccm) at 600 °C for 40 min under the pressure of 200–300 Pa. Similarly, Al–N-co-doped ZnO nanorods can also be obtained by putting the Al-doped ZnO nanorods into the high-temperature annealing furnace with the NH_3_ (15 sccm) and Ar (140 sccm) at 600 °C for 40 min under the pressure of 200–300 Pa.

### 2.4. Sample Characterizations

The morphologies and microstructures of undoped and doped ZnO samples were investigated using scanning electron microscopy (SEM, JSM-7100F, JEOL, Tokyo, Japan) and transmission electron microscopy (TEM, JEM-2100, JEOL). The HR-TEM images of all samples were conducted using the JEOL JEM-2100 TEM. The samples were firstly dispersed in ethanol solution and then the solution was dropped onto the copper mesh. Finally, the copper mesh with dried samples was placed in the TEM devices. The crystallinities of the products were characterized by the X-ray diffractometer (XRD, D8 advance). All samples were placed in the XRD equipment with the scan rate of 2/min from 10° to 90°. The components of the products were analyzed by X-ray energy dispersion spectrometry (EDS, Oxford Ultim max, Oxford, UK) and X-ray photoelectron spectroscopy (XPS, Thermo Fisher Scientific escalab250×, Waltham, MA, USA). XPS was investigated by an Al-Kα monochromated X-ray beam with a chamber pressure of 7 × 10^−8^ Pa. The emission angle was 57°, and the spot diameter was 500 µm. The peak for C 1s at 284.8 eV was used for calibration.

### 2.5. Field Emission and Hydrogen Sensing Investigations

The field emission (FE) test was carried out by a high vacuum field emission test system (TSV-300HH) in a bipolar structure with the undoped and doped ZnO samples as the cathode and the stainless steel as the anode. The test area of all samples was 0.16 cm^2^, and the distance between the two electrodes was 200 μm. The tests were conducted using a Keithley 248 high-voltage (Cleveland, OR, USA) power supply to provide stable voltage and a Victor 86E Victory multimeter (Los Angeles, CA, USA) to monitor the transmitting current in real-time. The current data were acquired through the computer.

The preparation process of the sensors and the schematic diagram of the field emission hydrogen sensing test system are shown in Figure S1 (see Supplementary Materials). The hydrogen sensing test was investigated based on the gas adsorption-induced field emission enhancement effect [4,8]. Firstly, an external voltage is applied on the undoped and doped ZnO samples, generating a high FE current (typically 100 µA) for several minutes to degas the adsorbed gases from the surface of all samples. High-purity hydrogen (99.999%) was then introduced into the vacuum chamber to keep the test pressure in the range of 10^−7^ to 10^−3^ Pa. In order to obtain reliable and stable pressure sensing data, the normalized current I_N_, that is, the average current obtained at the end of every 10 s during a certain emission period, was used to evaluate the hydrogen sensing performance.

## 3. Results

### 3.1. Morphologic and Structural Characterizations of the Undoped and the Doped ZnO Samples

**Figure 2:**
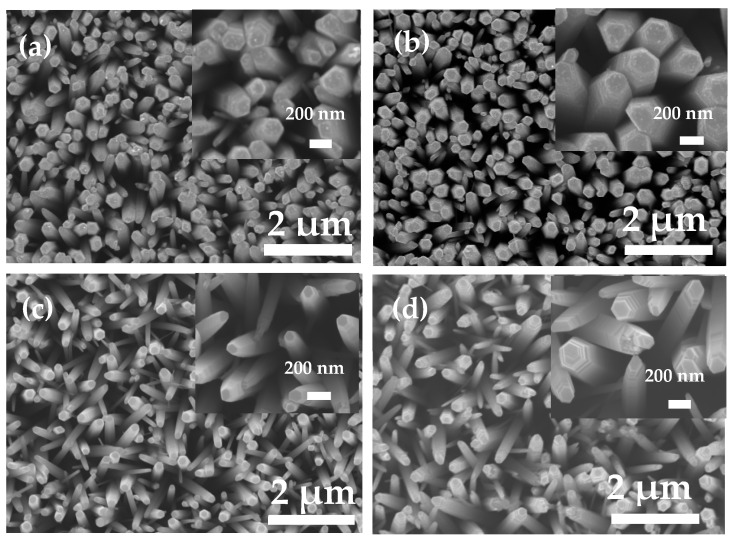
SEM images of the as-prepared ZnO-based samples. (**a**) undoped; (**b**) N-doped; (**c**) Al-doped; and (**d**) Al–N-co-doped ZnO samples.

Typical morphologies of the undoped, Al-doped, N-doped, and Al–N-co-doped ZnO nanorods are shown in Figure 2. As shown in Figure 2a, the undoped ZnO nanorods were rod-like structures with an average diameter of about 100–200 nm (see Figure S2 in Supplementary Materials). In contrast, the morphology and the diameter of N-doped ZnO nanorods (Figure 2b) did not show obvious difference, but the surface was partly corroded due to ammonia treatment for the N-doped ZnO. As shown in Figure 2c,d, it could be noticed that the average diameters of Al-doped and Al–N-co-doped ZnO nanorods were smaller than that of undoped ZnO nanorods, mainly due to the introduction of Al element. The activity of Al is higher than that of Zn, leading to easy binding with oxygen. In addition, the radius of Al^3+^ (0.535 Å) is smaller than that of Zn^2+^ (0.74 Å); therefore, the diameters and the corresponding tip curvatures of Al-doped and Al–N-co-doped ZnO nanorods are smaller [21]. The height of the undoped and doped ZnO nanorods is uniform due to the similar growth conditions. Furthermore, the densities of all samples per unit area are almost the same because the growth densities of all samples mainly depend on the number of zinc oxide seed particles per unit. All zinc oxide seed layers were obtained under the same conditions, thus leading to closed numbers of zinc oxide seed particles. For example, the numbers of zinc oxide nanorods for undoped, N-doped, Al-doped, and Al–N-co-doped samples are 47, 45, 43, and 44, respectively, under the same area of 4 µm^2^.

**Figure 3:**
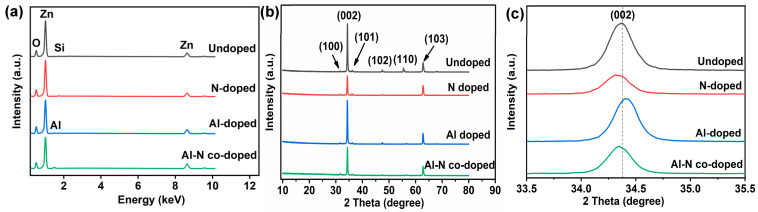
(**a**) EDS and (**b**,**c**) XRD results of undoped, Al-doped, N-doped, and Al–N-co-doped ZnO nanorods. Note: (**c**) is the enlargement part of 002 orientation from (**b**).

**Figure 4:**
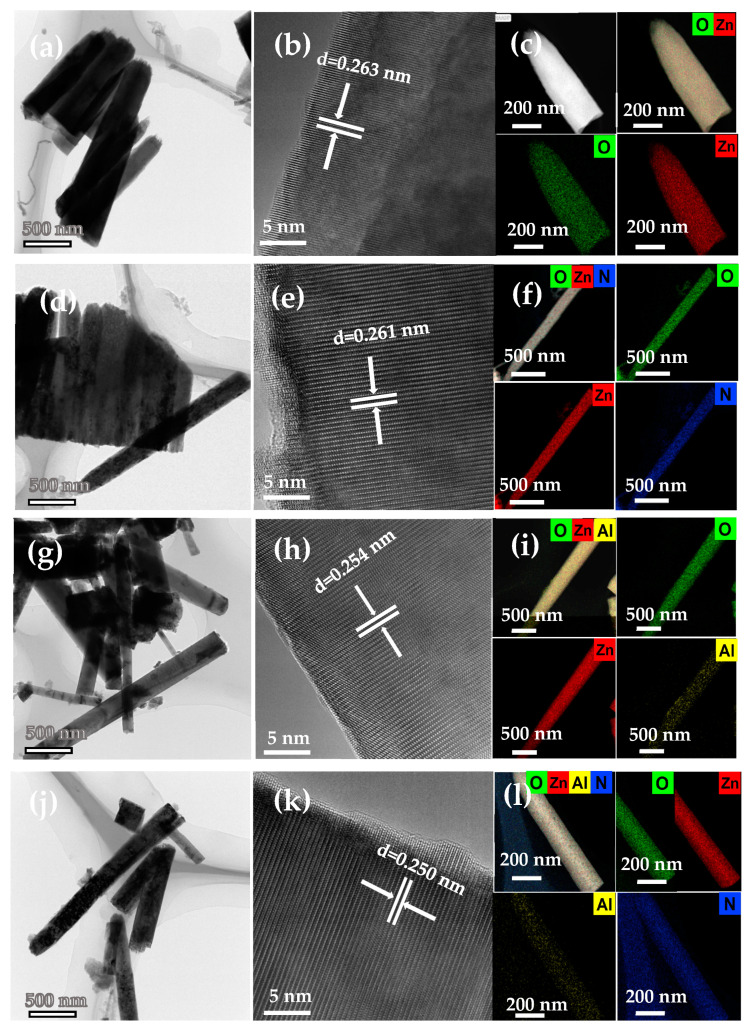
TEM images and EDS mapping analysis of the undoped and all doped ZnO nanorods. (**a**,**d**,**g**,**j**) low TEM images; (**b**,**e**,**h**,**k**) high-resolution TEM images; and (**c**,**f**,**i**,**l**) EDS mapping results for undoped, N-doped, Al-doped, and Al–N-co-doped ZnO samples, respectively.

EDS and XRD results of the undoped, Al-doped, N-doped, and Al–N-co-doped ZnO nanorods are shown in Figure 3. As shown in Figure 3a, the signals of Zn, O, and Si could be observed for undoped ZnO nanorods. The signals of Zn and O were from ZnO nanorods, and the Si signal was from the silicon substrate. Compared with the undoped ZnO nanorods, an additional signal of Al was observed in the Al-doped and Al–N-co-doped ZnO nanorods. However, the N element was not identified in the N doped and Al–N-co-doped ZnO nanorods, probably due to low contents of N beyond the instrument resolution for several reasons. Firstly, the N element is difficult to detect due to the low energy of the N element. Secondly, the characteristic X-ray wavelength of the N element is relatively short, and the N signal can be easily absorbed when escaping from the samples. Thirdly, the N element content is very low, and EDS could not detect it. From Figure 3b, the diffraction angles located at 31.6°, 34.4°, 36.2°, 47.6°, 56.5°, and 62.9° correspond to the (100), (002), (101), (102), (110), and (103) crystal faces, respectively, for all ZnO-based samples [22,23]. Additionally, the (002) crystal face exhibited the strongest peak among all samples, suggesting all the ZnO-based samples grew along the C-axis. Compared with the undoped ZnO samples, the dominant peak (002) of N-doped and Al–N-co-doped ZnO samples slightly shifted towards a lower diffraction angle, while the dominant peak of Al-doped ZnO sample shifted slightly towards a higher diffraction angle (Figure 3c), indicating that Al and N atoms were incorporated into the ZnO lattice [24,25,26].

(HR)TEM and EDS mapping images of the undoped, Al-doped, N-doped, and Al–N-co-doped ZnO nanorods are shown in Figure 4. The diameters of the undoped (Figure 4a) and N doped (Figure 4d) ZnO nanorods are about 200 nm, with the hexagonal wurtzite structure growing along (002) direction and the lattice spacing of 0.263 and 0.261 nm (Figure 4b,e), in agreement with the XRD results (Figure 3b). The components of the undoped (Figure 4c) and N-doped (Figure 4f) ZnO nanorod were characterized by EDS mapping, showing Zn, O, and N signals correspondingly. As shown in Figure 4g,j, the diameters of the Al-doped (Figure 4g) and Al–N-doped (Figure 4j) ZnO nanorods were ~150 nm with the hexagonal wurtzite structure growing along [0001] direction and the lattice spacing of 0.254 and 0.250 nm (Figure 4h,k), consistent with the XRD analysis (Figure 3b). The components of Al-doped (Figure 4i) and Al–N-doped (Figure 4l) ZnO nanorods were characterized by the EDS mapping, showing Zn, O, Al, and N signals correspondingly.

**Figure 5:**
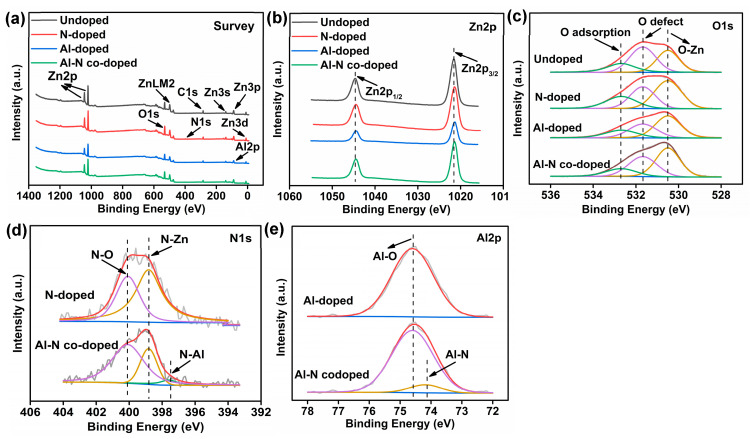
XPS spectra of the undoped, N-doped, Al-doped, and Al–N-co-doped ZnO samples. (**a**) Survey; (**b**) Zn2p; (**c**) O1s; (**d**) N1s; (**e**) Al2p.

In order to investigate the chemical state and the components, XPS characterizations were conducted for all samples with anticipated signals of Zn, O, N, and Al, as shown in Figure 5a. It could be noticed that the C peaks for all samples were observed due to the contamination from the XPS chamber [20]. For the undoped ZnO sample (Figure 5b), the Zn2p peaks at 1021.6 and 1044.6 eV correspond to Zn2p_3/2_ and Zn2p_1/2_ in the Zn–O bond, respectively [27]. But, the Zn2p peaks of all doped ZnO nanorods showed a slight shift towards low binding energies due to the incorporation of Al or N into the ZnO structure [27,28,29]. For the O1s spectra (Figure 5c), three peaks at 530.5, 531.7, and 532.7 eV could be assigned to the O–Zn bonds (lattice oxygen), the oxygen defects and the adsorbed oxygen, respectively [20,27]. For the N1s spectra (Figure 5d), two peaks at 398.3 and 400.1 eV corresponded to the N–Zn and N–O bonds with Zn–O–N state for the N-doped and Al–N-co-doped ZnO samples [30,31]. Besides, a minor peak at 397.6 eV might be assigned to the N-Al bond for the Al–N-co-doped ZnO sample [32]. For the Al2p spectra (Figure 5e), the dominated peak at 74.6 eV should be assigned to the Al-O bonds for the Al-doped and Al–N-co-doped ZnO samples. In addition, a minor peak at 74.2 eV should be assigned to Al–N bonds [33].

### 3.2. Field Emission Performance Test

**Figure 6:**
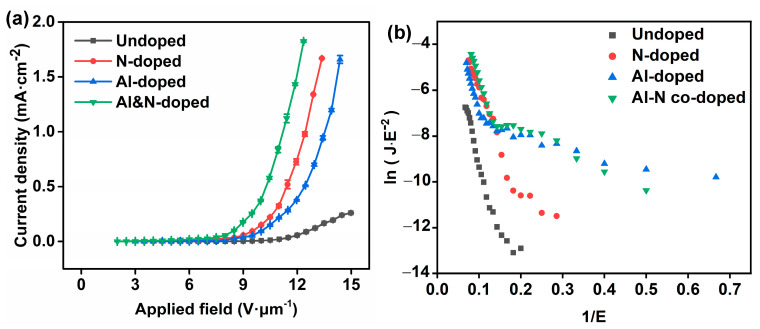
Field emission performances of undoped, Al-doped, N-doped, and Al–N-co-doped ZnO samples. (**a**) J–E curves and (**b**) the corresponding F–N plots of four samples.

**Figure 7:**
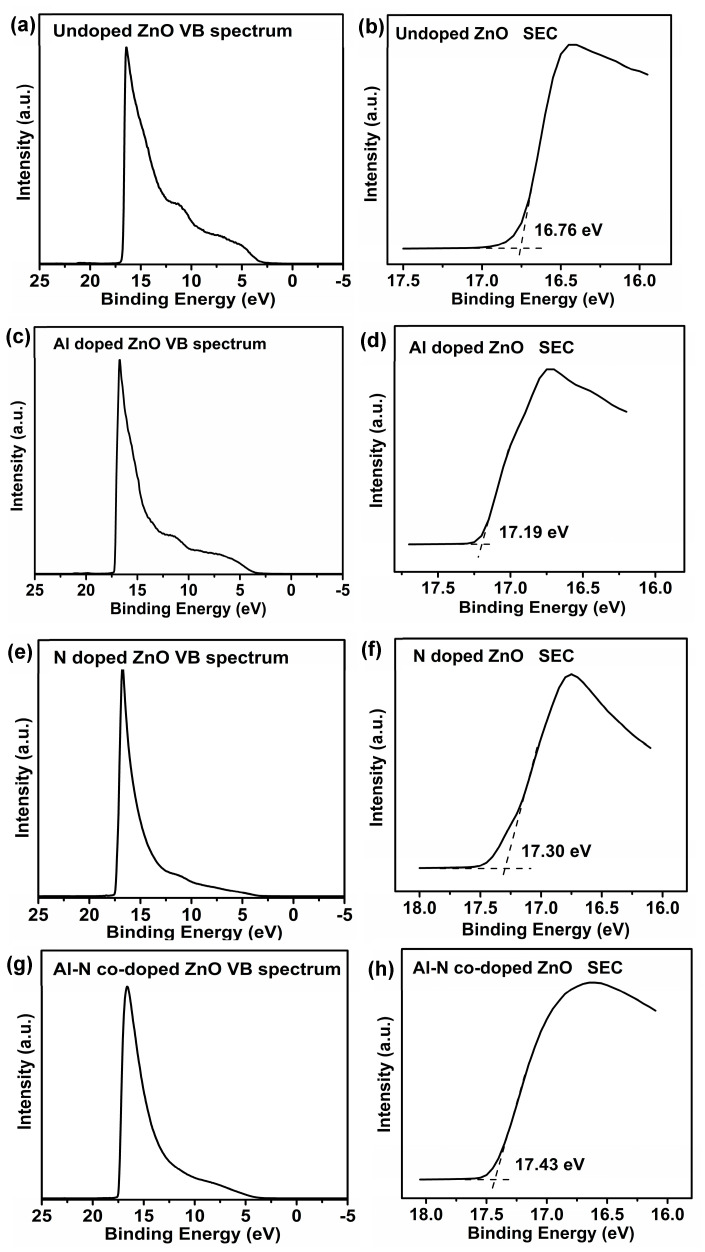
UPS measurements of the undoped, Al-doped, N-doped, and Al–N-co-doped ZnO samples. (**a**,**b**) undoped; (**c**,**d**) Al-doped; (**e**,**f**) N-doped; and (**g**,**h**) Al–N-co-doped ZnO samples. Note: SEC is the secondary electron cutoff, and VB is the valance band. (**b**,**d**,**f**) are the enlargement parts from (**a**,**c**,**e**).

The field emission curves of the undoped, Al-doped, N-doped, and Al–N-co-doped ZnO samples are shown in Figure 6 (Table 1). The turn-on field (E*_to_*) and the threshold field (E*_thr_*) are defined as the electric field to generate an emission current density of 10 μA·cm^−2^ and 1 mA·cm^−2^, respectively. As shown in Figure 6a, the turn-on field of the undoped, Al-doped, N-doped, and Al–N-co-doped ZnO nanorods were 9.9, 7.8, 6.9, and 5.0 V·µm^−1^, respectively. The threshold field of Al-doped, N-doped, and Al–N-co-doped ZnO nanorods were 13.5, 12.5, and 11.2 V·µm^−1^, respectively. In comparison with the undoped samples, all doped samples exhibited lower E*_to_* and E*_thr_*, attributed to the work function reductions (Figure 7). For the Al-doped and Al–N-co-doped ZnO, the larger aspect ratio can also decrease E*_to_* and E*_thr_* (see Figure S2 in Supplementary Materials). In addition, the doped ZnO samples exhibit lower resistances in comparison with the undoped ZnO sample [34,35,36], which might lead to the decrease of E*_to_* and E*_thr_*. Among doped ZnO samples, the Al–N-co-doped ZnO sample exhibited the best FE performance for its lowest WF and the larger aspect ratio (Figure 7 and Figure S2).

Two characteristic FE sections could be seen for all samples in Figure 6b due to the space charge effect, localized state, and gas adsorption [37,38,39,40,41,42,43]. Gas adsorbents might change the WFs of field emitters under different applied fields. The gas could adsorb on the surface of the samples at a low applied field, leading to the WF reduction but detaching from the surface at a high applied field, resulting in two FE states [41]. Therefore, in most cases, the slope of the FN curve at a high applied field is larger than that at the low applied field due to higher WF [40,41,43]. However, the FN curves in this study were the opposite, probably due to the surface states or shallow levels from the intrinsic defects of the samples [43]. Different defects in ZnO samples, e.g., Zn interstitials or oxygen defects, can be generated due to low oxygen concentration in the solution during the hydrothermal reaction [43], which was confirmed by the XPS results (Figure 5c).

To determine the work functions, UPS measurements were conducted on the undoped, Al-doped, N-doped, and Al–N-co-doped ZnO samples, as shown in Figure 7. The secondary electron cutoff (SEC) could be obtained by applying a bias of −5 V on all samples. In addition, the Fermi levels (EF) of all samples were calibrated with the value of 0 eV. Thus, the work functions could be calculated based on the following equation: ϕ = 21.22 − BE_SEC_ [8,44], where ϕ is the work function of the material, and BE_SEC_ is the binding energy of the secondary cutoff edge (SEC). Taking the undoped ZnO sample as an example (Figure 7a,b), the work function is 4.46 eV (21.22 − 16.76 = 4.46 eV). Similarly, the work functions for Al-doped, N-doped, and Al–N-co-doped ZnO samples could also be obtained with the values of 4.03, 3.92, and 3.79 eV, respectively.

### 3.3. Hydrogen Sensing Performance Test

**Figure 8:**
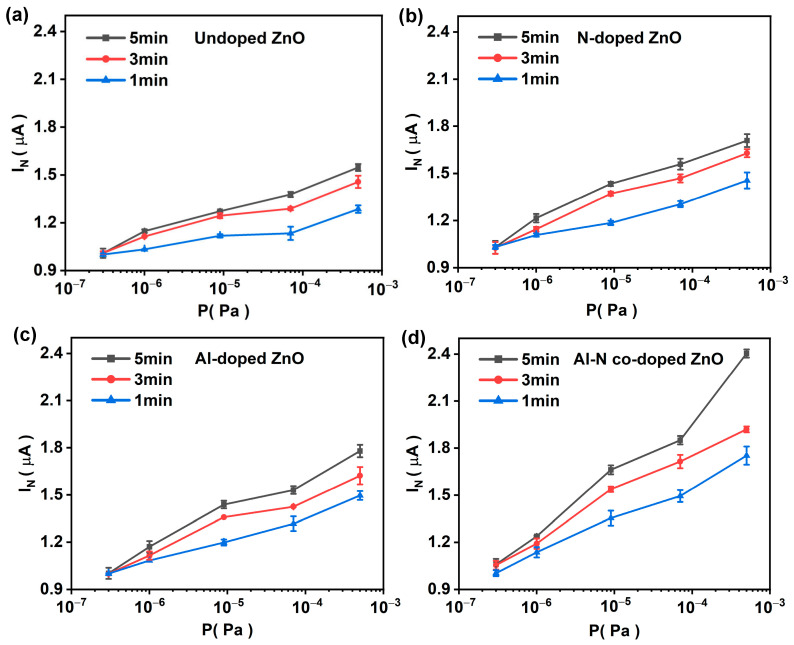
Pressure sensing performances for all samples. (**a**) undoped; (**b**) N-doped; (**c**) Al-doped; and (**d**) Al–N-co-doped.

As shown in Figure 8, the hydrogen sensing performance of four samples was investigated in the pressure range of 10^−7^~10^−4^ Pa under an initial emission current of 1.0 µA. In order to achieve high sensing reliability under low emission current conditions, the normalized average current I_N_ [45,46] was used (see Figure S3 in Supplementary Materials), and the pressure sensing curves of all samples with six tests on the same sample and six different samples and were obtained, showing good repeatability for these samples (see Figures S4 and S5 in Supplementary Materials). In addition, error bars associated with increasing I_N_ are required so as to claim better hydrogen sensing performance relative to other ZnO-based samples. As shown in Figure 8, with the increase of hydrogen pressure, the corresponding emission currents of the four ZnO samples increased, indicating that all samples have hydrogen sensing effects. Moreover, compared with the undoped ZnO sample (Figure 8a), all doped ZnO samples exhibited better sensing performance. For the doped ZnO samples, the increases of sensing current for Al-doped and N-doped ZnO samples can reach 71% and 78% at the test pressure of 5 × 10^−3^ Pa for 5 min emission, while the Al–N-co-doped ZnO sample exhibited the sensing current increase of 140% at the pressure 7 × 10^−3^ Pa for 5 min emission. In addition, the increase of sensing current for the Al–N-co-doped ZnO sample reached 75% at the pressure 7 × 10^−3^ Pa for 1 min emission, suggesting the Al–N-co-doped ZnO sample is more sensitive to low-pressure hydrogen sensing. The hydrogen adsorption causes the work function change of zinc oxide, leading to the hydrogen sensing effect. Specifically, hydrogen molecules first physically adsorb on the surface of ZnO nanomaterials, and then they are decomposed into H atoms through field emission Joule heating. The chemical adsorption of H atoms results in a decrease of the effective work function, leading to the increase of field emission current. This phenomenon is known as the low-pressure hydrogen sensing effect of ZnO, similar to carbon nanotubes reported before [4,46]. Compared with the undoped ZnO nanorod samples, the doped ZnO nanorods have a higher sensing current due to lower work function after hydrogen adsorption, as confirmed by the future work on Ni-doped ZnO nanowires by the first principles simulation. In comparison with the Pd–Au alloy and PdO nanomaterials, et al. [3,6,47,48], the Al–N-co-doped ZnO nanorods show better low-pressure sensing performances (See Table S1 in Supplementary Materials) with wide low-pressure range from 10^−7^ to 10^−4^ Pa, which is very significant for the hydrogen detection of the vacuum microelectronic devices. Further investigations should be focused on improving the response for quick hydrogen detection.

## 4. Conclusions

In summary, we report the low-pressure hydrogen sensing performance based on Al–N-co-doped ZnO nanorods with a test pressure of 10^−7^~10^−3^ Pa. The results suggest that the Al–N-co-doped ZnO is the most sensitive to low-pressure hydrogen sensing among all ZnO samples, with the highest sensing current increase of 140% for 5 min emission. In addition, the sensing current increase for the Al–N-co-doped ZnO sample could reach 75% at the pressure 7 × 10^−3^ Pa for 1 min emission. This work not only expands the hydrogen sensing applications to Al–N-co-doped ZnO nanomaterials but also provides a promising approach to develop a practical field emission cathode with a strong low-pressure hydrogen sensing effect. Meanwhile, this work indicates that other semiconductor oxide systems could be potential candidates for low-pressure hydrogen sensing applications.

## Figures and Tables

**Table 1:** Comparison of FE parameters of the undoped and the doped ZnO samples.

Sample	Turn-On Field (V/µm)	Threshold Field (V/µm)
Undoped ZnO	9.9	/
N doped ZnO	6.9	12.5
Al doped ZnO	7.8	13.5
Al–N-co-doped ZnO	5.0	11.2

## Data Availability

Data are contained within the article and Supplementary Materials.

## Supplementary Materials

The following supporting information can be downloaded at: https://www.mdpi.com/article/10.3390/nano14100863/s1, Figure S1. The schematic diagram of field emission hydrogen sensing test system. Figure S2. The diameter distributions of the undoped and the doped ZnO nanorods. Figure S3. The variation curves between the sensing current and the time under different partial pressure of hydrogen for Al-N co-doped ZnO nanorods. Figure S4. Pressure sensing performances for all samples with six tests on the same sample. (a–c) undoped; (d–f) N-doped; (g–i) Al-doped and (j–l) Al-N co-doped. Figure S5. The reproducible pressure sensing curves for six different Al-N co-doped ZnO samples under different test time. (a) 1 min; (b) 3 min and (c) 5 min. Table S1. Comparison of the low pressure sensing performances [3,6,47,48].
