# Supplementary material for: Enhanced Field Emission and Low-Pressure Hydrogen Sensing Properties from Al–N-Co-Doped ZnO Nanorods
Source: Nanomaterials (Basel). 2024 May 16;14(10):863. doi: 10.3390/nano14100863 (PMC11124068; doi:10.3390/nano14100863)
Supplement: Supplementary file 1 [file nanomaterials-14-00863-s001.zip › nanomaterials-2977810-supplementary.pdf]

Supplementary Material

Enhanced Field Emission and Low-Pressure Hydrogen Sensing Properties from Al–N-Co-Doped ZnO Nanorods

Youqing Tu, Weijin Qian*, Mingliang Dong, Guitao Chen, Youlong Quan, Weijun Huang and Changkun Dong*

Wenzhou Key Lab of Micro-Nano Optoelectronic Devices, Wenzhou University, Wenzhou 325035, China; YookingTu@163.com (Y.T.); dml13946319580@126.com (M.D.); 13124983550@163.com(G.C.); 17377260603@163.com (Y.Q.); 18857757816@163.com (W.H.); *Correspondence: weijinqian@wzu.edu.cn (W.Q.), dck@wzu.edu.cn (C.D.); Tel.: +86-577-86689067 (C.D.)

Index

SM-1. The schematic diagram of field emission hydrogen sensing test system

SM-2. Comparison of the diameter distributions of the undoped and the doped ZnO nanorods

SM-3. The variation curves between the sensing current and the time

SM-4. Pressure sensing performances for all samples with six tests on the same sample

SM-5. The reproducible data on six samples of sensor performances

SM-6. Comparison of the low pressure sensing performances

SM-1. The schematic diagram of field emission hydrogen sensing test system

We firstly prepared the undoped, Al doped, N doped, and Al-N co-doped ZnO samples. Then all test samples were placed in a high vacuum field emission testing system. The field emission was carried in a bipolar structure with the ZnO samples as the cathode and the stainless steel as the anode (Figure S1).

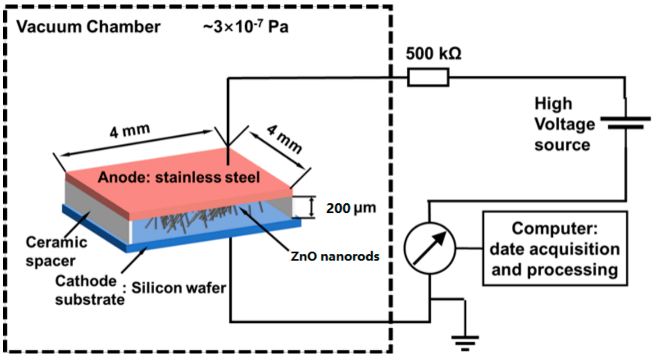

Figure S1. The schematic diagram of field emission hydrogen sensing test system

SM-2. Comparison of the diameter distributions of the undoped and the doped ZnO nanorods

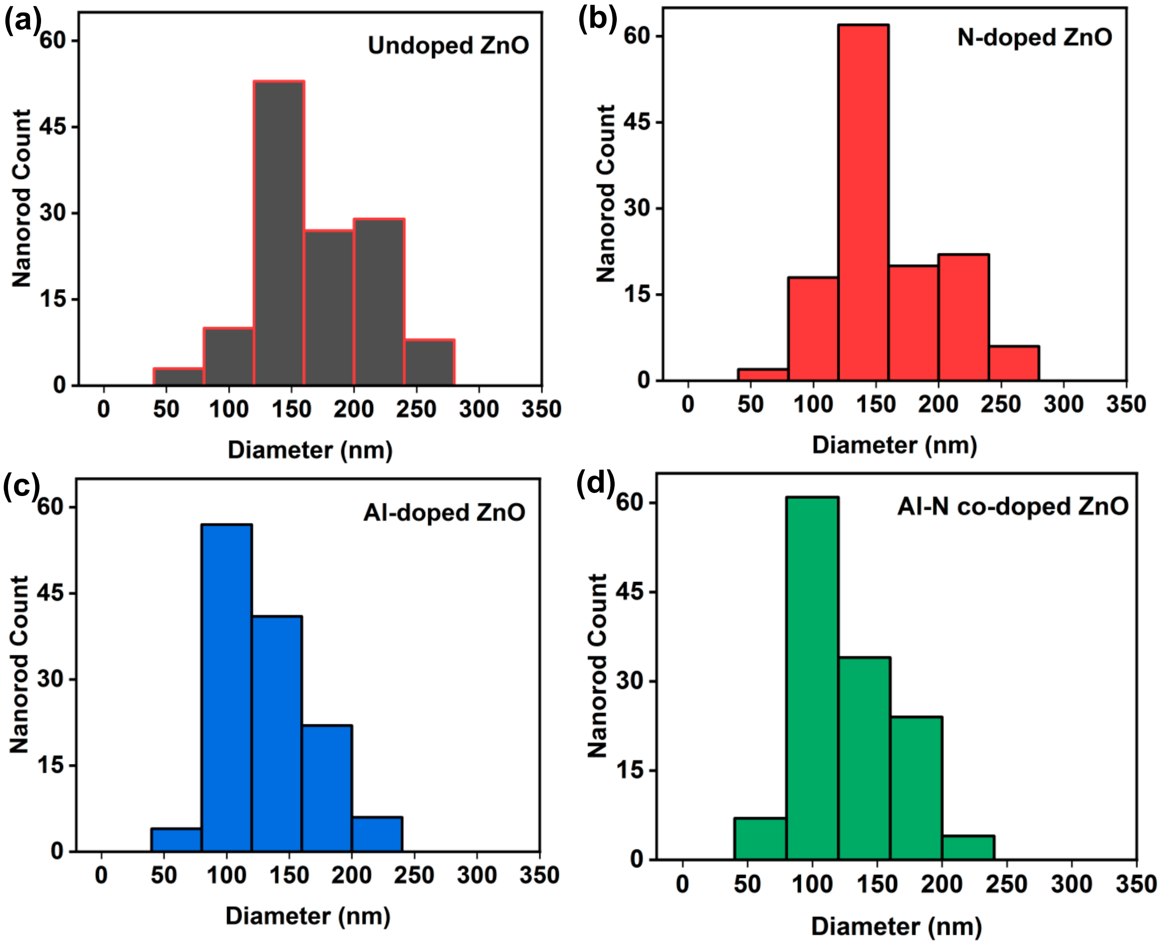

Figure S2. The diameter distributions of the undoped and the doped ZnO nanorods

The diameters of the undoped and N-doped ZnO nanorods are mainly in the range of 120 to 250 nm, While the diameter of Al doped and Al-N co-doped ZnO nanorods are mainly from 100 to 200 nm. For the samples of small diameters (less than 120 nm), the undoped and N-doped ZnO nanorods account for 10.0 and 15.4 percentages, respectively, while Al-doped and Al-N co-doped nanorods account for 46.9 and 54.6 percentages, respectively, suggesting larger aspect ratios.

SM-3. The variation curves between the sensing current and the time.

Taking Al-N co-doped ZnO nanorods for example, the field emission currents increased during a 5 min test period under a constant emission voltage, and the increase rates rose with increasing the pressure from 3×10^{-7} to 5×10^{-4} Pa. The variation curves between the sensing current and the time were firstly obtained by computer, as shown in Figure S3, then the normalized average current I_N was used to obtain the pressure sensing performance curves.

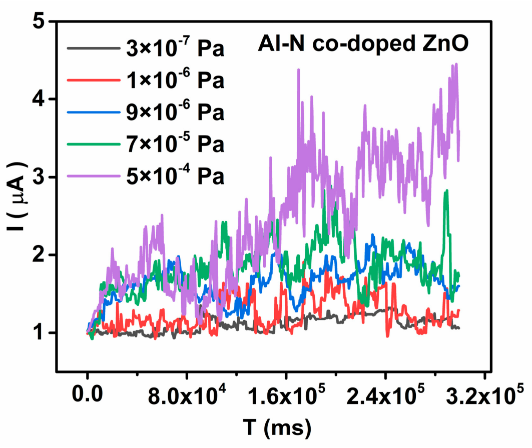

Figure S3. The variation curves between the sensing current and the time under different partial pressure of hydrogen for Al-N co-doped ZnO nanorods.

SM-4. Pressure sensing performances for all samples with six tests on the same sample

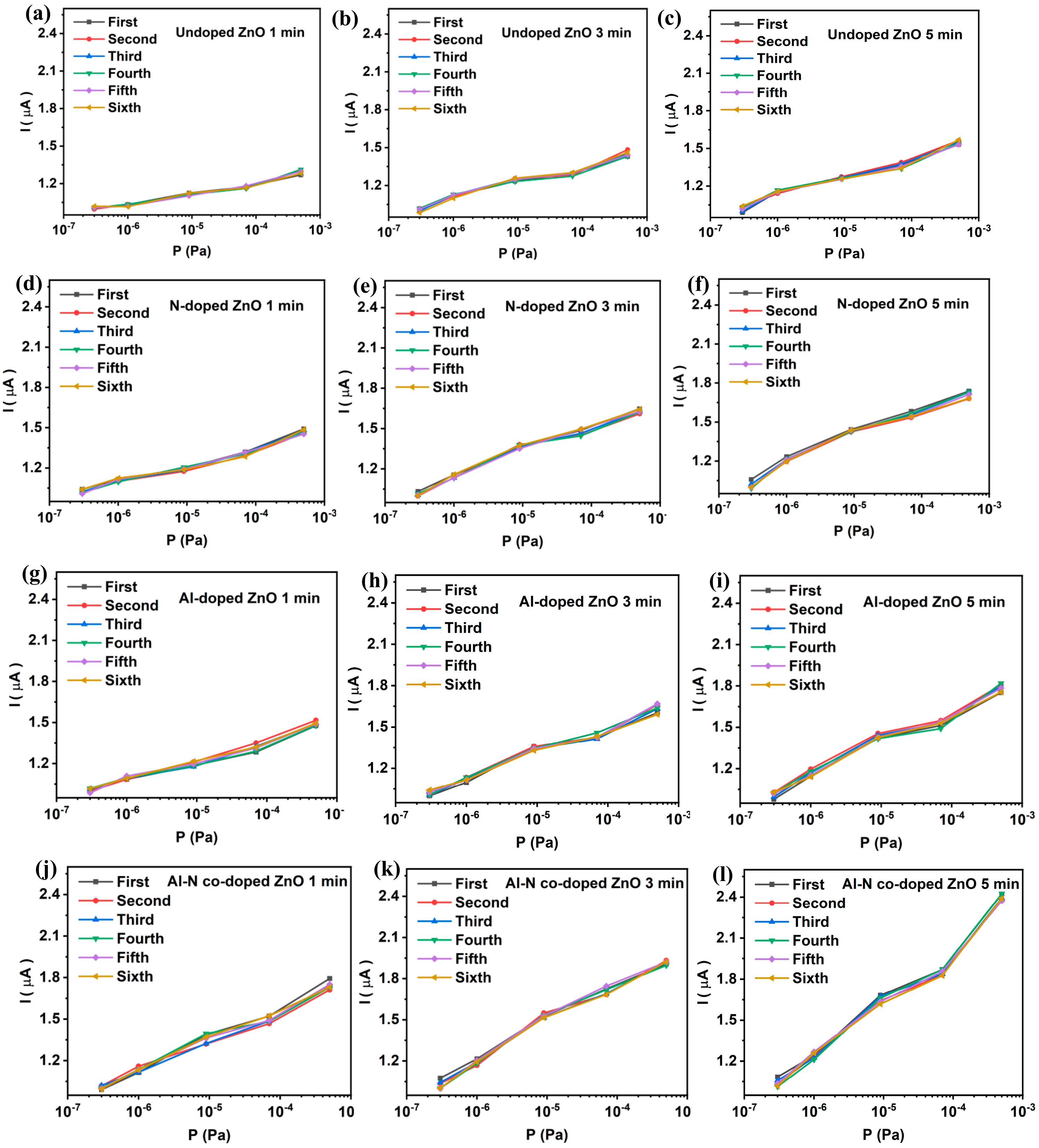

Figure S4. Pressure sensing performances for all samples with six tests on the same sample. (a-c) undoped; (d-f) N-doped; (g-i) Al-doped and (j-l) Al-N co-doped.

Multiple sensing tests on the same sample show good repeatability as shown in the Figure S4.

SM-5. The reproducible data on six samples of sensor performances.

The pressure sensing curves of six different samples were obtained, as shown in Figure S4, showing good repeatability for these samples.

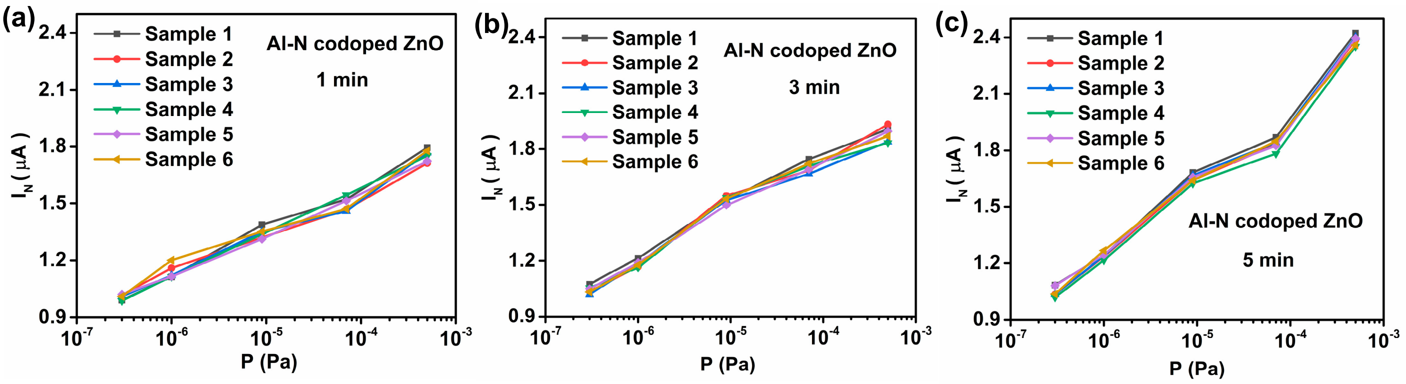

Figure S5. The reproducible pressure sensing curves for six different Al-N co-doped ZnO samples under different test time. (a) 1min; (b) 3min and (c) 5min.

SM-6. Comparison of the low pressure sensing performances

Table S1 Comparison of the low pressure sensing performances

Materials	Application	Detection range	Response time	References
Polymers	Electronic skin	10^{-2} - 10^{1} Pa	< 10 ms	[47]
NM-based vertical junction	Variable-area transport junction	10^{-2} - 10^{1} Pa	~ 80 ms	[48]
AuPd alloy	Nanoelectromechanical systems	10^{-3} -10^{-2} Pa	6.66 MHz	[3]
PdO nanoparticles	Plasma optical hydrogen sensor	1 mbar~1 bar	5 s	[6]
ZnO nanorods	Vacuum electronic device	10^{-7} - 10^{-4} Pa	60 s	This work
